# Draft Genome Sequence from a Putative New Genus and Species in the Family *M1A02* within the Phylum *Planctomycetes*, Isolated from Benthic Pinnacle Mats in Lake Untersee, Antarctica

**DOI:** 10.1128/mra.01192-21

**Published:** 2022-04-20

**Authors:** Nicole Yasmin Wagner, Dale T. Andersen, Aria S. Hahn, Ryan McLaughlin, Sarah Stewart Johnson

**Affiliations:** a Department of Biology, Georgetown University, Washington, DC, USA; b SETI Institute, Mountain View, California, USA; c Koonkie Inc., Menlo Park, California, USA; d Science, Technology, and International Affairs Program, Georgetown University, Washington, DC, USA; Universidad Nacional Autónoma de México

## Abstract

Here, we report the draft genome sequence for a new putative genus and species in the family *M1A02* within the order *Phycisphaerales*. Isolated from the metagenome of a benthic pinnacle-shaped mat in the Antarctic Lake Untersee, the members of this family have been found in biofilms and freshwater environments.

## ANNOUNCEMENT

Untersee is a perennially ice-covered lake in Antarctica (71°20′31.13″S, 13°27′16.45″E).

Benthic, photosynthetic microbial mats are present to depths exceeding 130 m. Small pinnacle mats up to 15 cm tall form at depths between 10 m and 15 m. Conical structures up to 70 cm tall form at depths between 10 m and 130 m (1, 2). Samples were obtained from pinnacle mats collected at 13 m using 50-mL Falcon tubes in fall 2019. The samples were preserved in 75% ethanol and later stored at −80°C. DNA was extracted using a Qiagen AllPrep kit (Germantown, MD). Library preparation was performed using the Illumina DNA-Prep Workflow (San Diego, CA) and run on the Illumina MiniSeq platform using a MiniSeq reagent midoutput (MO) kit (300 cycles) (Illumina Inc.), generating 151-bp paired-end reads. The reads were trimmed using Trimmomatic v0.39 (3). MetaWRAP v1.3 (4) was used to assemble and bin (using the binning module) the metagenomes into metagenome-assembled genomes (MAGs), with default parameters. In all, 38,626,094 reads were assembled, 3,740,790 of which mapped to the MAG described here.

This MAG was found in a pinnacle mat. It is 94.45% complete and 0.00% contaminated and has a GC content of 67%, as assessed using CheckM v1.1.3 (5) with default parameters. It contains 3,740,790 reads (2.51% of total metagenomic reads) in 59 contigs. An average coverage of 50.8× was calculated using BWA-mem2 v2.2.1 (6) and SAMtools v1.13 (7) with default parameters. The MAG comprises 3,099,537 bp with an *N*_50_ value of 132,036 bp. There are 2,454 open reading frames (ORFs). The MAG contains 127 tRNA genes and 1 small subunit 16S rRNA gene.

Using GTDB-tk v1.5.0 (8) with default settings, the MAG was classified as belonging to the family *M1A02*, in the order *Phycisphaerales*, phylum *Planctomycetes*. The closest full-length 16S match on NCBI (https://www.ncbi.nlm.nih.gov/), according to a Web-based BLASTN search of the NCBI nonredundant/nucleotide (nr/nt) database (accessed 11 January 2021) (9, 10), was a partial rRNA of an uncultured bacterium discovered in the northern South China Sea, with a 92% identity match (11), below the minimum identity (94.5%) used for 16S genus identification (12), suggesting that this MAG represents a new genus.

**FIG 1:**
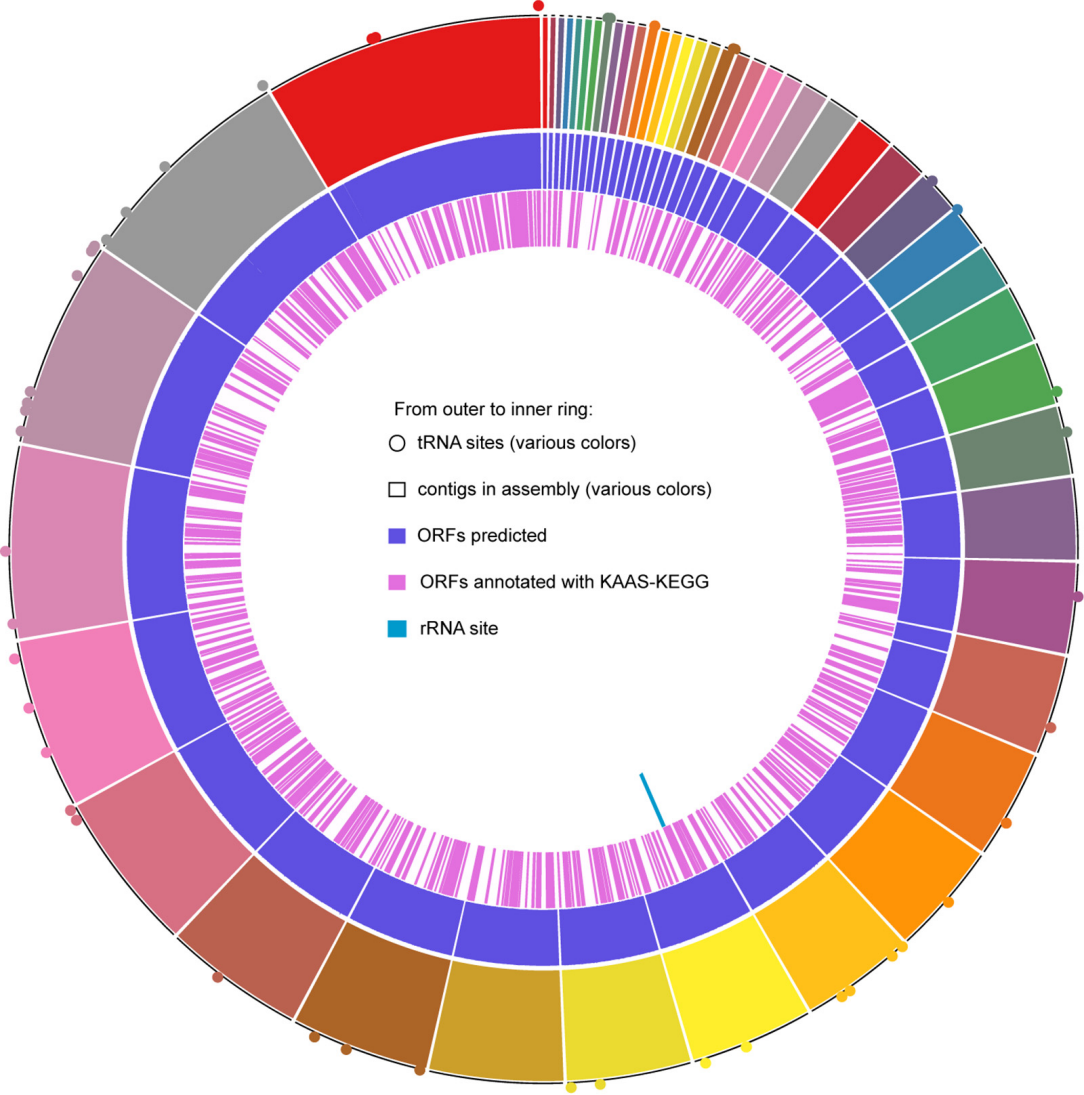
Metagenome-assembled genome (MAG) Circos plot showing the following (from outer to inner rings): first, tRNA locations; contigs based on their order in the MAG; the proportion of total predicted open reading frames (ORFs) (any sequence with a start and stop codon, excluding rRNA genes) with a corresponding annotation in the KEGG database (5); and finally, in the innermost ring, the location of the rRNA.

Functional annotation was performed using KAAS-KEGG v2.1 (13) with default parameters. A total of 18.13% of the ORFs code for nucleotide, amino acid, and lipid metabolism, 23.8% for carbohydrate and energy metabolism, and 32.36% for genetic information processing (Fig. 1). It contains *ppdK* used in the C4 photosynthesis pathway, which is known to increase the efficiency of carbon dioxide fixation in photosynthetic organisms and facilitate ATP production from glycolysis (14, 15). This MAG contains MDH1 and MDH2, present in the C4 photosynthetic pathway (16). Together, this evidence suggests that this new genus is capable of photosynthetic carbon cycling.

We identified ORFs (e.g., *cheR*, *cheB*, *cheA*, *cheW*, *oppA*, *oppB*, and *oppC*) responsible for chemotaxis and quorum-sensing (17), as well as biofilm formation, and a chemosensory system that regulates biofilm formation (*gacA*/*gacS* system and *wspR* [18, 19]). Additionally, we found ORFs related to the formation of flagellum (*fliF*, *fliG*, *fliM*, *fliN*, *flhA*, *flhB*, *motA*, *motB*, and *glfK*), suggesting motility (20, 21), and the presence of quorum-sensing and chemotaxis genes, suggesting that the MAG aids in pinnacle mat formation.

### Data availability.

This whole-genome shotgun project has been deposited at DDBJ/ENA/GenBank under accession number JAJOLK000000000. This study represents version JAJOLK010000000. The reads were deposited under BioProject accession number PRJNA784630. The GFF and gene and protein fasta files are accessible at https://doi.org/10.6084/m9.figshare.19376819.v1, and the functional annotation can be found at https://doi.org/10.6084/m9.figshare.19287740.v1.
